# RNA-binding proteins in human oogenesis: Balancing differentiation and self-renewal in the female fetal germline

**DOI:** 10.1016/j.scr.2017.04.008

**Published:** 2017-05

**Authors:** Roseanne Rosario, Andrew J. Childs, Richard A. Anderson

**Affiliations:** a MRC Centre for Reproductive Health, Queen's Medical Research Institute, University of Edinburgh, 47 Little France Crescent, Edinburgh EH16 4TJ, UK; b Department of Comparative Biomedical Sciences, The Royal Veterinary College, London NW1 0TU, UK

**Keywords:** Germ cell differentiation, RNA binding proteins, LIN28, DAZL, BOLL, FMRP

## Abstract

Primordial germ cells undergo three significant processes on their path to becoming primary oocytes: the initiation of meiosis, the formation and breakdown of germ cell nests, and the assembly of single oocytes into primordial follicles. However at the onset of meiosis, the germ cell becomes transcriptionally silenced. Consequently translational control of pre-stored mRNAs plays a central role in coordinating gene expression throughout the remainder of oogenesis; RNA binding proteins are key to this regulation. In this review we examine the role of exemplars of such proteins, namely LIN28, DAZL, BOLL and FMRP, and highlight how their roles during germ cell development are critical to oogenesis and the establishment of the primordial follicle pool.

## Highlights

• RNA-binding proteins (RBPs) are key regulators of gene expression during oogenesis.
• RBPs LIN28, DAZL, BOLL and FMRP display stage-specific expression in fetal oocytes.
• LIN28 and DAZL may regulate self-renewal and progression into meiosis respectively.
• BOLL and FMRP may be involved in the later stages of prophase I and oocyte growth.
• RBPs may have critical roles in establishing the ovarian reserve during fetal life.

## 1 Introduction

The finite nature of human female fertility is underpinned by the formation of a non-renewable reserve of primordial follicles that are assembled from mid-gestation onwards in humans (reviewed in (Findlay et al., 2015)). Establishment of the ovarian reserve begins with the migration of primordial germ cells (PGCs) from the proximal epiblast to the genital ridge; a process already underway in the human embryo at four weeks of development (Witschi, 1948, Mollgard et al., 2010), and which is largely complete by the eighth week of gestation (6 weeks post conception) (De Felici, 2013). Upon arrival at the gonad, and following female sex specification, PGCs undergo three significant, overlapping and possibly interconnected processes on their journey to becoming functional oocytes, namely: the initiation of meiosis, the formation and breakdown of germ cell nests, and the assembly of single oocytes into primordial follicles. It is these follicles which constitute the ovarian reserve for the adult life of women, and the developmental events prior to, and during their foundation, that lay the foundations of developmental competence required to form an oocyte that is capable of fertilisation in adult life.

### 1.1 Forming follicles

The formation of primordial follicles begins around 16 weeks gestation in humans (Motta et al., 1997, Bendsen et al., 2006), as nests of interconnected germ cells break down, releasing individual oocytes to associate with somatic pre-granulosa cells to form primordial follicles. The germ cell nest is an evolutionarily conserved structure, found in males and females from *Drosophila* (de Cuevas et al., 1997) and *Xenopus*, to mice (Pepling et al., 1999) and humans (Motta et al., 1997, Gondos et al., 1971). Nests form as a result of incomplete cytokinesis during germ cell mitosis, leading to the formation of a clonal syncytium of germ cells that divide synchronously and share cytoplasm (Grive and Freiman, 2015). Organelles are exchanged between interconnected germ cells in nests, and their distribution is reorganised just prior to nest breakdown in mice (Pepling and Spradling, 2001), a process linked to the selection of a single oocyte (Lei and Spradling, 2016). Nest breakdown is a coordinated effort involving the loss of germ cells through caspase-dependant apoptosis and physical invasion of the nests by somatic cells (Tingen et al., 2009). It is estimated that up to two-thirds of all germ cells are lost during nest breakdown (Pepling and Spradling, 2001). This culling of germ cells may represent a means of germ cell selection, through which deficient cells are lost and only the highest quality oocytes are assembled into primordial follicles.

**Fig. 1:**
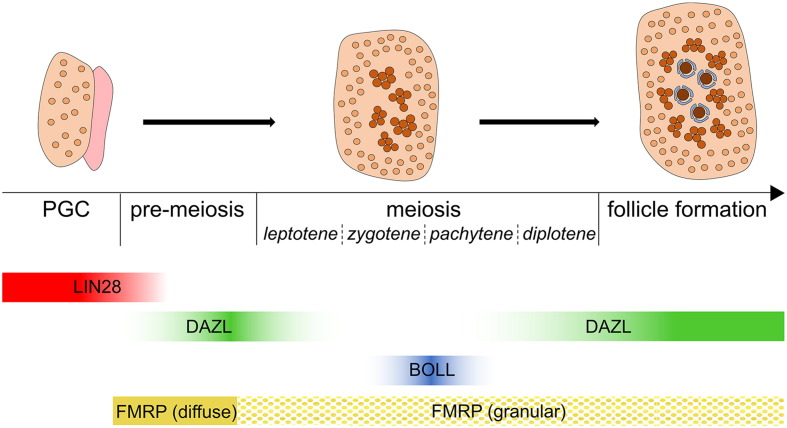
LIN28, DAZL, BOLL and FMRP expression during germ cell differentiation in females. Cartoon schematic depicts spatial and temporal organisation of germ cells within the human fetal ovary. Germ cells at different stages of maturation are represented by progressively darker shades of orange. LIN28 is present in PGCs. DAZL is expressed before the onset of meiosis but down-regulated afterwards; BOLL is transiently expressed at later stages of meiosis with minimal overlap with DAZL. DAZL is re-expressed in oocytes within primordial follicles. FMRP is present in pre-meiotic germ cells, and yellow dots represent granulation of FMRP staining at the onset of meiosis (Section 2.4).

In humans, the first primordial follicles to form are located deep within the centre of the fetal ovarian medulla, whilst undifferentiated, mitotic germ cells, with characteristics of PGCs, are found towards the periphery of the ovary (Fig. 1). The human fetal ovary shows distinct spatial and temporal organisation, with more differentiated germ cells found progressively deeper into the ovary, establishing a distinct developmental gradient (Anderson et al., 2007, Childs and Anderson, 2012). Thus, the entire developmental spectrum from PGC to primordial follicle can be observed on a single histological section by 18 weeks' gestation, providing an excellent developmental paradigm in which to study the process of cellular differentiation (He et al., 2013a). Similar processes occur in the sheep and cow (Sawyer et al., 2002, Hummitzsch et al., 2013). This cortico-medullary gradient of increasing germ cell differentiation differs from that of the fetal mouse ovary, in which differentiation proceeds in an anterior to posterior (Menke et al., 2003, Bullejos and Koopman, 2004) and possibly dorsal-ventral (Cordeiro et al., 2015) wave along the gonadal axis. Why such differences exist is not clear, but may reflect the need to maintain niches for undifferentiated, proliferating germ cells, which persist alongside more differentiated meiotic germ cells and follicular oocytes in the developing ovaries of larger mammals (Fereydouni et al., 2014, Fulton et al., 2005). In contrast, in the ovaries of feto-neonatal rodents (Kimura et al., 2011), germ cell proliferation is largely complete before the major wave of follicle assembly commences. Despite these differences, however, the assembly of the first follicles occurs at the centre of the developing ovary in both humans and mice, suggesting some aspects of the spatio-temporal regulation of germ cell differentiation may be conserved (Mork et al., 2012, Zheng et al., 2014).

### 1.2 Meiosis

The initiation of meiosis is one of the defining features of germ cell differentiation, and occurs during fetal life in females, as opposed to from puberty in males. Although comprised of two rounds of cell division, only prophase of meiosis I occurs during fetal oogenesis, with arrest occurring before completion of the first division. The timing of meiotic entry is not intrinsic to germ cells themselves, but rather depends on exposure to retinoic acid produced by the mesonephros in rodents (Bowles et al., 2006, Koubova et al., 2006), but probably by the fetal ovary itself in humans (Childs et al., 2011, Le Bouffant et al., 2010, Bowles et al., 2016, Frydman et al., 2017).

Following pre-meiotic DNA replication, germ cells within nests enter leptotene of prophase I and initiate recombination by generating double strand DNA breaks (Roig et al., 2004, Baudat et al., 2013), leading to the pairing and synapsis of homologous chromosomes during zygotene. The synaptonemal complex, which holds synapsed chromosomes together, is assembled by pachytene, and throughout zygotene and pachytene, meiotic recombination generates crossovers, which not only increase genetic diversity, but also provide physical connections that keep homologous chromosomes together once the synaptonemal complex dissociates in diplotene (Petronczki et al., 2003, MacLennan et al., 2015). Following diplotene, the oocytes enter a period of meiotic (dictyate) and growth arrest, and the nests of interconnected oocytes break down, releasing individual oocytes to form primordial follicles. The oocytes are then maintained in this arrested state until oocyte growth is initiated, a hiatus that can extend to decades in humans. Although oocyte growth occurs throughout follicle development, meiosis only recommences at the time of ovulation. During this prolonged period in stasis, cohesion proteins are important in maintaining the physical linkage between sister chromatids, and deterioration in chromatid cohesion contributes significantly to age-dependent aneuploidy (Jessberger, 2012, Herbert et al., 2015).

Whether germ cell nest breakdown and primordial follicle formation are tied to proper meiotic progression remains unclear. Depletion of synaptonemal complex protein 1 (Sycp1) in fetal rat ovaries (to accelerate the onset of diplotene) resulted in primordial follicles being assembled earlier and in greater numbers than in control ovaries, suggesting an intricate relationship between diplotene arrest and primordial follicle formation (Paredes et al., 2005). However, the ovaries of *Stra8*^−/−^ mice (in which germ cells fail to initiate meiosis) contain ‘oocyte-like’ cells and follicular structures, suggesting that meiosis and oogenesis/follicle formation may be uncoupled, although the failure of such oocyte-like cells to support development confirms that meiosis is essential to confer reproductive potential (Dokshin et al., 2013, Baltus et al., 2006).

## 2 RNA-binding proteins in fetal oogenesis

Mammalian gametogenesis, and particularly oogenesis, is punctuated by periods of transcriptional silencing, during which homeostasis and development are dependent on the translation of pre-transcribed mRNAs, under the regulation of RNA-binding proteins (RBPs) (Clarke, 2012, Seydoux and Braun, 2006, Radford et al., 2008). RBPs are an extensive class of proteins, defined by their ability to recognise particular motifs and bind RNA *via* specific recognition sites usually found in 3′ untranslated regions (3′UTRs). RBPs found in the cell nucleus primarily govern nascent mRNA (pre-mRNA) processing events (capping, polyadenylation and splicing), whilst those located in the cytoplasm are known to regulate translation by directing mRNA transport and regulating mRNA stability (Brook et al., 2009). Importantly, RBPs are highly expressed during oogenesis and have been well documented as being an essential component of post-transcriptional control during all stages of germ cell development. Animal knockout models of germ cell-expressed RBPs often exhibit various stages of developmental arrest during gametogenesis and resultant infertility (Ruggiu et al., 1997, VanGompel and Xu, 2010, Tay and Richter, 2001). Therefore research surrounding the mechanisms utilised by RBPs during germ cell development is critical to our overall understanding of oogenesis and the establishment of the ovarian reserve. In this review we examine the role of such RBPs, specifically LIN28, DAZL, BOLL and FMRP, in initiating and sustaining germ cell development in the human fetal ovary (Fig. 1), and highlight recent findings made by ourselves and others in this regard.

### 2.1 LIN28: balancing oogonial differentiation and self-renewal?

**Fig. 2:**
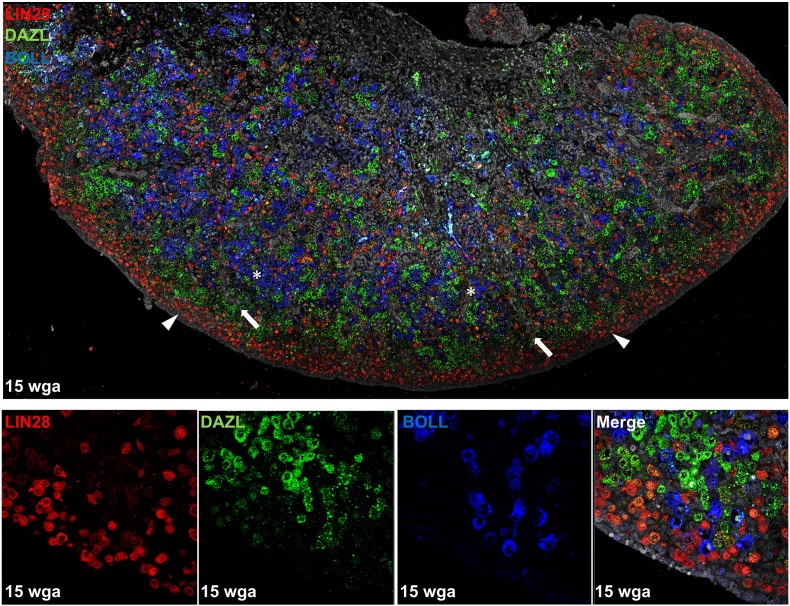
LIN28, DAZL and BOLL expression in human fetal ovary. Tiled image of 15wga human fetal ovary section depicts LIN28 positive germ cells (red) in the periphery of the ovary (arrowheads), whilst DAZL positive germ cells (green) are more mature and located further from the ovary edge (arrows). BOLL positive germ cells (blue) are more centrally located and have a larger diameter size (asterisks). There is no co-localisation between LIN28, DAZL or BOLL (as observed in merge image).

The RNA-binding protein LIN28 is a critical regulator of cellular pluripotency, differentiation, survival and homeostasis across a diverse range of tissues (Shyh-Chang and Daley, 2013). Lin28 is required for normal specification of the PGC population (West et al., 2009), and *Lin28*^−/−^ mice have reduced germ cell numbers at e13.5 and birth, and form fewer primordial follicles (Shinoda et al., 2013). We, and others, observed developmentally-regulated expression of *LIN28* in the human fetal ovary, with expression decreasing with increasing gestation (Childs et al., 2012, El-Khairi et al., 2012). Consistent with PGC-specific expression of Lin28 in the mouse, we found LIN28 to be expressed exclusively by germ cells in the human fetal ovary (Fig. 2), and restricted to primordial and premeiotic germ cells (Childs et al., 2012). We observed no change in expression across gestation of the paralogous gene *LIN28B*, which has been implicated in the pathogenesis of ovarian cancer (Permuth-Wey et al., 2011) and the timing of menarche (Perry et al., 2009, Ong et al., 2009). The cell type(s) expressing LIN28B in the human fetal ovary remain to be determined (Childs et al., 2012, El-Khairi et al., 2012).

In addition to its pluripotency-associated role in ES cells, Lin28 is also required for the maintenance of tissue-specific progenitor cells in the developing embryo (Urbach et al., 2014, Yang et al., 2015), and for the differentiation of the first germ layers in *Xenopus* embryos (Faas et al., 2013), suggesting roles for LIN28 in the regulation of differentiation as well as the maintenance of stem cell identity (Tsialikas and Romer-Seibert, 2015). Consistent with this, we noted that LIN28 expression persists beyond that of the pluripotency-associated transcription factor OCT4/POU5F1 in human fetal germ cells, but is extinguished before the onset of meiosis (as evidenced by the absence of SYCP3 and LIN28 co-expression) (Childs et al., 2012). Together, these data indicate that LIN28 may have regulate both the maintenance of undifferentiated PGCs and the earliest steps of oogenesis, following commitment to differentiation and the loss of ‘stemness’ in PGCs.

Although LIN28 can regulate translation of target mRNAs through direct binding, it also acts to antagonise the activity of the *let-7* family of microRNAs (Shyh-Chang and Daley, 2013, Huang, 2012). In the human fetal ovary *let-7* microRNA expression mirrors that of LIN28 (Childs et al., 2012), suggesting that the high levels of LIN28 in undifferentiated germ cells may be required to restrict high levels of *let-7* transcripts at this stage. Indeed, the balance of these factors in the fetal germline seems critical, as overexpression of *let-7* in fetal mouse ovaries recapitulates the infertility phenotype seen in Lin28-deficient mice (Shinoda et al., 2013). Conversely, elevated LIN28 levels in human germ cell tumours (GCTs) correlate with decreased *let-7* microRNA levels, and increased expression of oncogene mRNAs subject to negative regulation by *let-7* (such as *MYCN*) (Murray et al., 2013), which in turn may contribute to the molecular pathogenesis of GCTs, and indeed other cancers (Molenaar et al., 2012, Powers et al., 2016). Human GCTs are widely thought to arise from germ cells that aberrantly retain, or re-acquire, the expression of PGC ‘stem cell’ markers, thus a role for LIN28 in the pathogenesis of GCTs reinforces the case for this protein being involved in balancing self-renewal and/or differentiation in human fetal germ cells. Intriguing recent data suggests that both the self-renewal and meiotic differentiation programmes may be activated simultaneously in pre-invasive male GCT cells (known as carcinoma *in situ* (CIS) (Jorgensen et al., 2013). It is therefore tempting to speculate that tighter regulation of the transition from self-renewal to meiotic differentiation in the fetal female germ cell may underpin the profound differences in the frequency of GCTs between males and females (GCTs account for 98% of testicular, but only 2–3% of ovarian cancers) (Bosl and Motzer, 1997, Permuth-Wey and Sellers, 2009). Close examination of the molecular phenotype and dynamics of germ cell proliferation and differentiation in the *Lin28*^−/−^ fetal mouse ovary may provide further insight into this.

### 2.2 DAZL: gatekeeper of meiosis?

*DAZL* (deleted in azoospermia-like) encodes a protein which belongs to the DAZ family of RBPs along with homologue members *DAZ* and *BOLL* (previously known as *BOULE*; discussed below in Section 2.3). DAZL contains two functional domains: a highly conserved RNA recognition motif (RRM) and a single DAZ domain (of unknown function) unique to DAZ family proteins. Only found in vertebrates, *DAZL* is specifically expressed in germ cells at all stages of oogenesis. In the human fetal ovary, *DAZL* transcript and protein expression increases sharply between 9 weeks (first trimester) and 14 weeks (early second trimester) gestation (Anderson et al., 2007, He et al., 2013b). This increase in expression immediately precedes the onset of meiosis. During this period, DAZL protein undergoes a shift in localisation from the nucleus (where it may have a role in RNA processing and storage) to the cytoplasm, where it is expected to function as a regulator of translation (Anderson et al., 2007, Reynolds and Cooke, 2005). Such a transition has also been observed in human fetal and adult mouse testis, where it coincides with the differentiation of fetal gonocytes and spermatogonia, respectively (Reijo et al., 2000). This would suggest that the relocalisation of DAZL protein is indicative of a potential shift in function between pre-meiotic and meiotic germ cells.

DAZL expression is subsequently down-regulation as meiosis progresses, but is re-expressed in oocytes of newly-formed primordial follicles (He et al., 2013b). How this biphasic pattern of DAZL expression is achieved remains unclear, but it bears a striking resemblance to that of KIT. In the human fetal ovary (and that of other species) KIT is expressed in pre/early-meiotic germ cells, downregulated during progression through meiotic prophase, and then re-expressed by oocytes assembled into primordial follicles (Robinson et al., 2001, Hoyer et al., 2005, Hutt et al., 2006). Whether KIT signalling has a role in regulating DAZL expression is unknown, but this raises a broader question of how RBPs in germ cells act as effectors of the plethora of growth factors to which germ cells are exposed (and about which we known very little). Dazl expression persists through later stages of oocyte maturation including through to zygote formation, where Dazl-dependent translation is thought to be necessary for spindle assembly, the metaphase I-II transition and early embryo development (Chen et al., 2011).

As previously mentioned, the timing of meiotic entry in germ cells is dependent upon exposure to retinoic acid. However, DAZL plays an important role in this, as it acts as a meiotic competence factor, enabling germ cells to respond to the meiosis-inducing signal. In the absence of DAZL, germ cells fail to develop beyond the PGC stage (shown by continued expression of pluripotency markers), giving rise to the concept that DAZL is a ‘licensing factor’ required for meiotic entry (Gill et al., 2011). Furthermore, the germ cell-specific expression of Dazl distinguishes germ cells from ovarian somatic cells (Lin et al., 2008), thus ensuring only the former respond to retinoic acid by entering meiosis. Retinoic acid induces the expression of the gene *Stra8* (*Stimulated by retinoic acid 8*), which in turn is required for the first critical steps of meiosis (Anderson et al., 2008). Germ cells in Dazl-deficient ovaries have significantly reduced expression of Stra8, which suggests that Dazl has an essential function upstream of meiotic initiation (Lin et al., 2008). Furthermore, as Dazl is not abundantly expressed in migrating PGCs, this prevents germ cells from responding to retinoic acid they might encounter during their migration to the genital ridge (Spiller et al., 2012). Upon reaching the gonad Dazl expression is activated, permitting germ cell responsiveness to cues from the somatic environment (Feng et al., 2014). However, the mechanism by which Dazl achieves this is currently unclear.

The phenotype of Dazl deficiency has been studied in detail in mice. *Dazl*^−/−^ mice are infertile due to defects in germ cell differentiation and a failure to progress beyond leptotene of meiotic prophase I (Ruggiu et al., 1997, Gill et al., 2011, Haston et al., 2009, Schrans-Stassen et al., 2001). *Dazl*-null gonads of both sexes also show a loss of post-migratory PGCs, although the severity of this is highly variable between individual animals (Saunders et al., 2003). Indeed, the overall phenotype of Dazl deficiency is more consistent and pronounced in inbred C57BL/6 mice (Lin and Page, 2005) than in non-inbred mice (Ruggiu et al., 1997, Schrans-Stassen et al., 2001, Saunders et al., 2003). For example, studies conducted in mice of a mixed genetic background suggest that Dazl is essential for the development of XY germ cells only after birth, yet on an inbred (C57BL/6) background, male Dazl^−/−^ mice lose their germ cells as early as e14.5, with the requirement for Dazl manifesting itself around the time that germ cells lose pluripotency and commit to a spermatogenic fate (Lin and Page, 2005).

If genetic heterogeneity is the cause of this variability in *Dazl*^−/−^ mice phenotypes (Saunders et al., 2003), this raises the question as to whether genetic background may also influence our understanding of *DAZL*-deficiency phenotypes in humans. Some evidence suggests this in fact is the case: deletion of *DAZ* gene(s) results in highly variable testicular defects ranging from complete germ cell absence, spermatogenic arrest with formation of few spermatids or severe oligozoospermia (Reijo et al., 1995, Reijo et al., 1996). Furthermore, single nucleotide polymorphisms in *DAZL* have been correlated with total sperm count, sperm motility, age at menopause and primary ovarian insufficiency (POI), in infertile men and women, respectively (Teng et al., 2002, Tung et al., 2006). However these studies were carried out in Asian populations, and attempts to replicate these findings in Caucasian populations have been unsuccessful (Bartoloni et al., 2004, Zerbetto et al., 2008).

The majority of current evidence points towards DAZL being an enhancer of translation. DAZL is associated with actively translating polysomes (Maegawa et al., 2002, Tsui et al., 2000) and sucrose gradient analysis of translation intermediates revealed Dazl specifically stimulates translation through regulation of the initiation stage (Collier et al., 2005). Furthermore in mouse, Dazl has been shown to stimulate the translation of Mvh, Sycp3 and Tex19.1 mRNAs (Chen et al., 2011, Reynolds et al., 2007, Reynolds et al., 2005). Therefore Chen et al. (2014) were surprised to find Dazl interacting with RNA processing bodies in mouse PGC-like cells (derived from mouse embryonic stem cells), as these structures are widely known to be involved in translational repression (Filipowicz, 2005, Liu et al., 2005). Using RNA immunoprecipitation and microarray a panel of Caspase mRNAs, namely Caspase 2, 7 and 9, were identified as Dazl targets, and loss of Dazl expression released *Caspase7* translational inhibition, thereby causing PGCs to enter apoptosis (Chen et al., 2014). This may provide a mechanism by which germ cells are lost by apoptosis in the Dazl^−/−^ testis (Lin and Page, 2005). Also co-immunoprecipitated with Dazl were mRNAs important for the maintenance of pluripotency in embryonic stem cells (*Sox2* and *Sall4*) as well as mRNAs required for differentiation of pluripotent cells (*Suz12*) (Chen et al., 2014), and these too were repressed by Dazl.

The SOX family of transcription factors are involved in maintenance of pluripotency, and in early human germ cells SOX17, rather than Sox2 in the mouse, is present (de Jong et al., 2008, Perrett et al., 2008). *In vitro* work from our own laboratory has identified *SOX17* as another pluripotency marker that is inhibited by DAZL in the human fetal ovary (unpublished data). Endogenous *SOX17* expression and *SOX17*-3′UTR *luciferase* translation decreased following overexpression of DAZL in HEK293 cells due to direct interaction between DAZL and the 3’UTR of *SOX17*. At 65 days gestation there was significant overlap between SOX17 and DAZL expression in germ cells in the human fetal ovary, however at 14 weeks gestation, after meiosis has commenced, SOX17 is predominantly found in less mature DAZL negative PGCs. Therefore through translational regulation of these specific RNAs, DAZL also limits both the pluripotency programme and somatic differentiation in nascent PGCs.

Recent work within our own laboratory has made efforts to expand the current knowledge regarding RNA targets of mammalian DAZL important for germ cell maturation. Thus far, attempts to identify DAZL targets have been mainly focussed on mouse, and the majority of studies have used germ cells isolated from the testis (refer to (Rosario et al., 2016a) for a review of RNA targets of DAZL). To analyse DAZL targets during the onset and early stages of meiosis, we carried out RNA sequencing of transcripts immunoprecipitated with endogenous DAZL from human fetal ovarian tissue. Our data confirm the meiotic role of DAZL in the human fetal ovary, and also reveal novel potential functions for DAZL through translation regulation of RNA targets involved in chromosome cohesin establishment (*SMC1B*) and recombination and DNA repair (*HORMAD1*, *TRIP13*, *TEX11*, *RAD18*, *RAD51*) (Rosario et al., 2017). Although these functions were also identified by a gene expression analysis of the *Dazl*^−/−^ mouse fetal ovary (Soh et al., 2015), we have extended this by using a variety of translational techniques to confirm the dependency on DAZL for translation of three specific RNAs: *SYCP1*, *TEX11*, and *SMC1B*. Therefore, we suggest DAZL has a key role in regulating fundamental processes that are responsible for aiding differentiating germ cells, through repression of pluripotency factors and initiation of meiosis.

### 2.3 BOLL: bridging prophase and primordial follicle formation

*BOLL* is considered to be the common ancestor of the DAZ family RBPs, yet despite this the physiological role of mammalian *BOLL* was the last to be explored. *BOLL* is strongly conserved evolutionarily, with orthologues in nearly all metazoans. Sequencing of the human *BOLL* gene in 200 fertile and infertile men revealed few sequence variants (Xu et al., 2003, Westerveld et al., 2005), in comparison to human *DAZL*, which has common variants at approximately 1 in every 100 basepairs (Teng et al., 2002, Tung et al., 2006). Such a high level of sequence conservation in a reproductive gene would suggest that BOLL has an essential germ cell role in animals.

In *Drosophila* males (Eberhart et al., 1996) and *C. elegans* females (Karashima et al., 2000), mutations in *boule* lead to meiotic arrest during pachytene of prophase I. Human BOLL (BOULE-like) has also been implicated in meiosis as it is able to restore meiotic function in *Drosophila boule* mutants (Xu et al., 2003). Unexpectedly, targeted disruption of *Boll* in mice revealed that Boll is not essential for the completion of meiosis, but is still required for the subsequent differentiation of round spermatids into mature spermatozoa (VanGompel and Xu, 2010). Boll null female mice showed no obvious defects and were fertile, therefore it appears the requirement for BOLL is specific only to male germ cells, much like the male-specific requirement of *DAZ* in humans (VanGompel and Xu, 2010), although ectopic expression of BOLL in human ES cells enhanced the differentiation of female cells into PGCs (Kee et al., 2009).

Prior to our work investigating BOLL expression in the human and mouse fetal ovary, the only report of BOLL protein in ovaries was in *C. elegans* (Maruyama et al., 2005). We found *BOLL* mRNA to be absent in first trimester fetal ovaries (which contain only premeiotic germ cells), but readily detectable in early second trimester ovaries, consistent with the entry of germ cells into meiosis at this time (He et al., 2013b). This is consistent with data from the fetal sheep ovary, in which *DAZL* transcript expression precedes that of *BOLL* by several days (Mandon-Pepin et al., 2003). We found BOLL protein to be expressed exclusively by oocytes in the human fetal ovary, and co-expression analysis revealed that a much greater proportion of BOLL-expressing cells also expressed meiosis markers (SYCP3 or phosphoATM) than DAZL-expressing cells. This indicates that human fetal oocytes switch from expressing DAZL to expressing BOLL early in meiotic prophase. As oocyte differentiation progresses, BOLL is subsequently downregulated, and DAZL re-expressed, around the time of germ cell nest break down and primordial follicle formation (Fig. 2) (He et al., 2013b). It therefore seems that DAZL is required for the initiation and early stages of germ cell differentiation and entry into meiosis, whilst BOLL may required for meiotic differentiation to be sustained once underway. Whether downregulation of BOLL – and reactivation of DAZL – is required for follicle formation remains to be established.

We also re-examined the expression of Boll in the fetal mouse ovary, and detected a similar pulse of Boll expression at e15.5. However unlike in humans where little overlap between BOLL and DAZL expression was observed, in mice Boll expression occurred in germ cells that also expressed Dazl, indicating that the two proteins are co-expressed in germ cells in the fetal mouse germline (He et al., 2013b). This overlapping expression raises the possibility of functional redundancy occurring between the two proteins during oogenesis in the mouse, and is likely to explain why *Boll*^−/−^ female mice are fertile. Furthermore, the absence of such co-expression (and thus lack of redundancy) in the human fetal oocyte raises the possibility that *BOLL* may be novel locus for human female (in)fertility, and suggests that some functional activities of DAZL and BOLL may have diverged between humans and mice. Decreased BOLL expression has been reported in infertile men with spermatogenic failure, however no mutations or polymorphisms were identified in *BOLL* which could explain this, suggesting that the spermatogenic failure must arise from factors upstream of BOLL (Lin et al., 2009, Luetjens et al., 2004). Whilst *Cdc25* (*Twine*) has been identified as a Boule target in *Drosophila* (Maines and Wasserman, 1999), almost nothing is known about the mRNA targets of mammalian BOLL, but the presence of conserved residues in the RNA-binding domains (Jenkins et al., 2011), plus the ability of BOLL to rescue Dazl-deficient phenotypes in flies and mouse (Xu et al., 2003, Vogel et al., 2002), suggests some conservation of targets with DAZL.

### 2.4 FMRP: setting up the oocyte for later development?

**Fig. 3:**
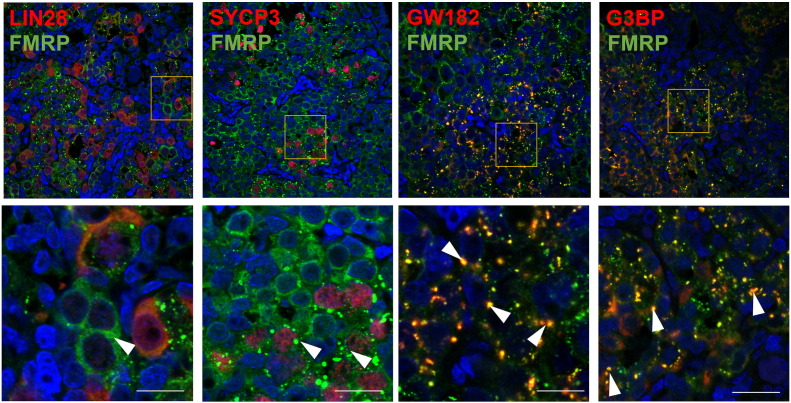
FMRP co-expression with various markers of germ cell development and RNA granules. There is limited overlap between FMRP and LIN28. Expression of the meiosis marker SYCP3 correlates with FMRP granulation (white arrowheads) in most but all of germ cells, suggesting that FMRP granulation precedes SYCP3 expression. There is a degree of association between FMRP and RNA granule markers GW182 and G3BP, respectively.

An intriguing story is emerging regarding the role of another translational repressor RBP, Fragile X Mental Retardation Protein (FMRP), in human oogenesis. Expansion of a CGG trinucleotide repeat in the 5’UTR of the *FMR1* gene (encoding FMRP) beyond 200 repeats results in fragile X syndrome, but around 20% of women with 55–200 repeats (known as premutation alleles) display a condition known as fragile X-associated premature ovarian insufficiency (FXPOI) (Sherman, 2000, Sherman et al., 2014, Mailick et al., 2014). To explore whether this infertility defect has its origins in fetal oocyte development, we investigated the distribution of FMRP in the human fetal ovary. Whilst initially diffusely distributed throughout the cytoplasm in mitotic PGCs, FMRP displays a striking redistribution into granular aggregates, which occurs coincident with, or immediately prior to, the initiation of meiosis (Fig. 3) (Rosario et al., 2016b). In neurons, FMRP is a component of several different ribonucleotide particle (RNP)-containing granules and within these granules, FMRP associates with its target mRNAs to control their subsequent storage, translation or degradation (Siomi et al., 1993, Hinds et al., 1993). Co-expression analyses with markers of known granule types to revealed that FMRP-rich foci associated with components of stress granules and P-bodies in the human fetal ovary (Fig. 3) (Rosario et al., 2016b). However the localisation of FMRP granules to germ cell cytoplasm leads to the attractive hypothesis that these structures are in fact, a class of germ cell granule, as these share many components with stress granules and P-bodies, including GW182 (Kotaja et al., 2006). Genetic evidence from several models supports an essential role for germ cell granules during germ cell differentiation: in the female mouse germline P-body-like granules are found in meiotically-immature oocytes (Aravin et al., 2009, Suzuki et al., 2007, Flemr et al., 2010), and a loss of RNAs found in these granules causes failure of primordial germ cell migration, reduced germ cell proliferation, pre-meiotic germ cell death, and meiotic defects (Kobayashi et al., 1996, Subramaniam and Seydoux, 1999, Koprunner et al., 2001, Carmell et al., 2007, Deng and Lin, 2002, Tanaka et al., 2000). However, the molecular mechanisms that underlie germ cell granule involvement in these phenotypes are unclear and mammalian germ cell granules remain poorly understood. Nevertheless, the appearance of these granules at the onset of meiosis in the human fetal ovary raises the possibility that FMRP is repressing mRNAs whose translation must be silenced to allow the mitosis-meiosis transition.

Mouse models engineered to carry *Fmr1* pre-mutation alleles (Hinds et al., 1993, Hoffman et al., 2012), show normal numbers of primordial follicles, suggesting that the initial stages of meiotic prophase and follicle formation are not perturbed by the presence of these alleles. Whether this is due to an intrinsic abnormality within the oocytes or pre-granulosa cells of primordial follicles, or in the pathways that control follicle activation is unclear, as is its possible relevance to human primordial follicle formation and oocyte development (Alvarez-Mora et al., 2015). Several RNA targets of FMRP have been identified in human brain, and work in the *Fmr1* null mouse, which exhibits precocious follicular activation, has demonstrated dysregulation of these targets in *Fmr1*^−/−^ ovaries, indicating that FMRP targets share signalling pathways across different cellular contexts (Ascano et al., 2012). Therefore it will be interesting to establish whether FMRP granule assembly is disrupted in FXPOI patients, and whether this has later impacts later oocyte/follicle development in these individuals, thus contributing to their subfertility.

## 3 Conclusions

Whilst it is clear that RNA-binding proteins play crucial roles in regulating oogenesis, understanding the functions of these proteins relies on identifying the RNA targets they bind and regulate. Given the relative promiscuity of RNA-binding proteins, it seems likely that the fertility phenotypes seen in their absence may not be attributable to dysregulation of a single mRNA target, but the combination of a failure to appropriately translate or process many transcripts. In addition to RNA-binding proteins, long non-coding RNAs (lncRNAs) have emerged as key regulators of pluripotency, differentiation, gene expression and chromatin structure and remodelling in mammalian cells, and can act as molecular sponges, to ‘soak up’ and inhibit the activity of miRNAs (Rosa and Ballarino, 2016). Each of these processes are critical to gametogenesis, yet we know little of the expression and/or function of such transcripts in the developing mammalian germline (Taylor et al., 2015). Finally, the recent identification of Pumilio 1 as a somatic cell-expressed RNA-binding regulator of oogenesis and follicle formation (Mak et al., 2016), underlines the need to broaden studies of the role(s) of RBPs in gametogenesis beyond the germ cell compartment alone. Single cell RNA sequencing studies, coupled with the development of new mouse models that enable stage-specific conditional deletion of RNA-binding proteins or that recapitulate infertility-associated polymorphisms in humans, will illuminate these issues.

## Funding

The authors' work in this field has been supported by grants from the Medical Research Council (G1100357 to RAA), Medical Research Scotland (354FRG to AJC), and the Royal Society (RG140503 to AJC).
